# Detection and differentiation of herbicide stresses in roses by Raman spectroscopy

**DOI:** 10.3389/fpls.2023.1121012

**Published:** 2023-06-05

**Authors:** Charles Farber, Madalyn Shires, Jake Ueckert, Kevin Ong, Dmitry Kurouski

**Affiliations:** ^1^ Department of Biochemistry and Biophysics, Texas A&M University, College Station, TX, United States; ^2^ Department of Plant Pathology and Microbiology, Texas A&M University, College Station, TX, United States; ^3^ Department of Biomedical Engineering, Texas A&M University, College Station, TX, United States; ^4^ Department of Molecular and Environmental Plant Science, Texas A&M University, College Station, TX, United States

**Keywords:** Raman spectroscopy, herbicides, roses, PLS-DA, glyphosate, Weed-B-Gon

## Abstract

Herbicide application is a critical component of modern horticulture. Misuse of herbicides can result in damage to economically important plants. Currently, such damage can be detected only at symptomatic stages by subjective visual inspection of plants, which requires substantial biological expertise. In this study, we investigated the potential of Raman spectroscopy (RS), a modern analytical technique that allows sensing of plant health, for pre-symptomatic diagnostics of herbicide stresses. Using roses as a model plant system, we investigated the extent to which stresses caused by Roundup (Glyphosate) and Weed-B-Gon (2, 4-D, Dicamba and Mecoprop-p (WBG), two of the most commonly used herbicides world-wide, can be diagnosed at pre- and symptomatic stages. We found that spectroscopic analysis of rose leaves enables ~90% accurate detection of Roundup- and WBG-induced stresses one day after application of these herbicides on plants. Our results also show that the accuracy of diagnostics of both herbicides at seven days reaches 100%. Furthermore, we show that RS enables highly accurate differentiation between the stresses induced by Roundup- and WBG. We infer that this sensitivity and specificity arises from the differences in biochemical changes in plants that are induced by both herbicides. These findings suggest that RS can be used for a non-destructive surveillance of plant health to detect and identify herbicide-induced stresses in plants.

## Highlights

- We report an innovative laser-based approach for non-invasive diagnostics of herbicide damage in plants.
- Our Raman-based technique enables ~90% accurate detection of Roundup- and WBG-induced stresses one day after application of these herbicides on plants.
- Our results also show that the accuracy of diagnostics of both herbicides at seven days reaches 100%.

## Introduction

Application of herbicides is a cornerstone of modern horticulture. However, many ornamental plant species are sensitive to herbicides and are commonly damaged from non-target exposure by mechanisms such as drift (Henry et al., 2004; Mehdizadeh et al., 2021). Moreover, herbicide-induced damages in some plant species, such as roses (*Rosa* spp.), have visual similarities with the symptoms induced by biotic stresses. For instance, chlorosis of leaves and shortened internodes caused by herbicide application on roses is often misdiagnosed as Rose Rosette Disease (RRD), a devastating disease that affects plants in Europe and the U.S (Farber et al., 2019a). Substantial biological and horticultural expertise is required to detect and identify symptoms of herbicide exposure; such expertise typically requires years of experience and training. Therefore, it becomes very important to develop a confirmatory analytical method that can be used to detect symptoms of herbicide exposure, as well as disentangle biotic and abiotic stresses in ornamental plants (Tataridas et al., 2022).

Roundup and WBG are some of the most used herbicides worldwide. In the US alone, between 1974 and 2014, the use of herbicides with glyphosate as their active ingredient, such as Roundup, increased almost 200-fold, from 635,000 kg to 125 billion kg (Benbrook, 2016). Glyphosate acts by inhibiting 5-enolpyruvylshikimate-3-phosphate (EPSP) synthase, a key enzyme in the shikimate pathway, which is responsible for synthesis of aromatic amino acids in plants (Sherwani et al., 2015). The effect of glyphosate is primarily evident at the sites of new growth (root and shoot meristem). Symptoms of glyphosate exposure, such as chlorosis and necrosis of tip tissues, can be observed across the entire plant at about 5 to 10 days after the herbicide application. Because products of the shikimate pathway are closely associated with a host defense, glyphosate also increases plant susceptibility to pathogens (Hammerschmidt, 2018)

While glyphosate has generic targeting, WBG is highly specific for dicots. WBG is a formulation of three different synthetic auxins: 2,4-dichlorophenoxyacetic acid (2,4-D), mercoprop-p acid (MCPP), and dicamba acid. Auxins are a family of plant hormones which are associated with regulating plant growth (Teale et al., 2006). The mechanisms of auxinic herbicides are not well understood (Kelley and Riechers, 2007; Mithila et al., 2011). Previous studies of these herbicides’ modes of action found that they trigger buildup of abscisic acid and ethylene, which enables accumulation of hydrogen peroxide, and subsequently, reactive oxygen species (ROS) (Romero-Puertas et al., 2004). Consequently, ROS are closely associated with the activity of WBG in plants (Gleason et al., 2011). A growing body of evidence suggests that several methods could be used detect herbicide stresses in plants, including hyperspectral imaging (Henry et al., 2017; Lu et al., 2020; Yan et al., 2021).

Our group previously showed that Raman spectroscopy (RS), a non-destructive, non-invasive analytical method that reveals the chemical structure of analyzed samples, can be used to detect and identify biotic and abiotic stresses in plants (Mandrile et al., 2019; Farber et al., 2020c; Gupta et al., 2020; Farber et al., 2021; Payne and Kurouski, 2021). The efficacy of this method has been demonstrated for the rapid detection of: viral diseases in roses, tomatoes, wheat, and ornamental shrubs; fungal diseases in corn, wheat, and sorghum; and bacterial diseases of orange (Yeturu et al., 2016; Egging et al., 2018; Farber and Kurouski, 2018; Farber et al., 2019a; Mandrile et al., 2019; Sanchez et al., 2019b; Farber et al., 2020a). Additionally, our group has demonstrated RS-based detection of insect larva developing within beans (Sanchez et al., 2019a) and the detection of zebra chip disease in potato (Farber et al., 2021). It was also reported that RS could be used for pre-symptomatic diagnostics of nutritional deficiencies in rise caused by the lack of nitrogen, phosphorus, and potassium (Sanchez et al., 2020).

Expanding upon this, we investigated the accuracy of RS-based confirmatory diagnostics of herbicide stresses in the ‘Pink Double Knock Out^©^’ roses that are caused by Roundup and WBG herbicides. Our findings suggest that RS can be used for a fast and accurate diagnostics of herbicide stresses in plants prior to the symptom development. Timely detection of such stresses can be used for timely elimination of the herbicides or adjustment of their concentrations.

## Materials and methods

### Plants

Twenty-one plants of the rose cultivar ‘Pink Double Knock Out^©^’ were used in this experiment. Plants were received as small tissue culture plants and were acclimated indoors for one month. After plants were actively growing, they were placed in a greenhouse to continue growing for 2 months before the experiment. The substrate used in the greenhouse was Jolly Gardener^®^ Pro-Line C/20, which consisted of 80% Canadian Sphagnum peat moss and 20% coarse perlite. We used neither fertilization nor pesticides before or during the experiments. The temperatures in the greenhouse were approximately 29°C during the day (6 am to 6 pm) and 21°C night (6 pm to 6 am). All plants involved in the experiment were screened for rose rosette virus (RRV), as well as for other rose viruses utilizing National Clean Plant Network-Rose screening protocols. All plants were free of the targeted pathogens (Farber et al., 2019a). Seven plants of uniform size and health were selected as replicates for each of the herbicide treatments.

### Herbicide treatments

The herbicides used for this experiment were Roundup and WBG; reverse osmosis (RO) water was used as the negative control application. The two herbicides were chosen due to the prevalence of those brands being used on lawns and in landscapes. To simulate herbicide drift damage, all herbicides were diluted to 1/10th of the Ready To Spray (RTS) amounts. This concentration was chosen to model appearance of symptoms that visually resembled RRD since such herbicide-induced stresses are often misdiagnosed as RRD in roses. Herbicide calculations were done based on a six-inch pot size. Roundup and WBG were diluted from the RTS at a rate of 1-part spray to 9 parts water. Application was performed by soaking all leaves to the point upon which solution droplets appeared on the plant surfaces.

### Raman spectroscopy

For each measurement, thirty leaflets were sampled randomly from each group of plants with an average of four leaves per plant. Leaflets were collected from both new, fully emerged leaves and mature leaves to represent the entire plant canopy. We avoided collection of leaflets that had any mechanical damage, signs of wilting, or leaves exhibiting visual signs of herbicide damage to demonstrate robustness of this sensing approach. All leaflets looked healthy without any changes in coloration or chlorosis symptoms. Sampling time intervals were: 1, 7, 14, and 30 day post-treatment. The experiment was repeated twice.

Raman spectra were acquired from leaves using an Agilent Resolve spectrometer equipped with an 830 nm source with a spectral resolution of 15 cm^-1^. Laser beam size was around 2 mm. For each measurement, the spectrometer was positioned next to the leaf surface. On average 2-3 spectra were collected form one leaf. All scans were taken in the ‘surface’ mode with a 1-second integration time and 490 mW of power. We found that as the experiment proceeded, the overall intensity of spectra acquired from herbicide-treated plants was much lower than that of the control plants. To address this, we normalized our spectra to the 1440 cm^-1^ peak. All spectral interpretation is based on these normalized spectra. All acquired spectra were baselined by the Agilent Resolve spectrometer. For the data analysis, Raman spectra were extracted using Agilent Resolve software and treated using MATLAB equipped with PLS-Toolbox ((Eigenvector Research Inc.). Reference Raman spectra of round-up and WBG are shown in the **Figure S1**.

### Statistical analysis

After spectra were imported into MATLAB and assigned a class based on their health or herbicide status, Partial Least Squares Discriminant Analysis (PLS-DA) was conducted to differentiate the spectra based on spectral changes associated with their experimenter-assigned classes. Spectra were first split into calibration (66%) and validation (34%) sets using the Kennard-Stone method before building all models, unless otherwise noted (Kennard and Stone, 1969). Spectra were normalized to a total spectral area of 1 then mean centered. All tables reporting PLS-DA results are for the validation of these models. All datasets used for model calibration and validation in this study are summarized in **Tables S1–S3**.

## Results

### Description of visual symptoms

**Figure 1:**
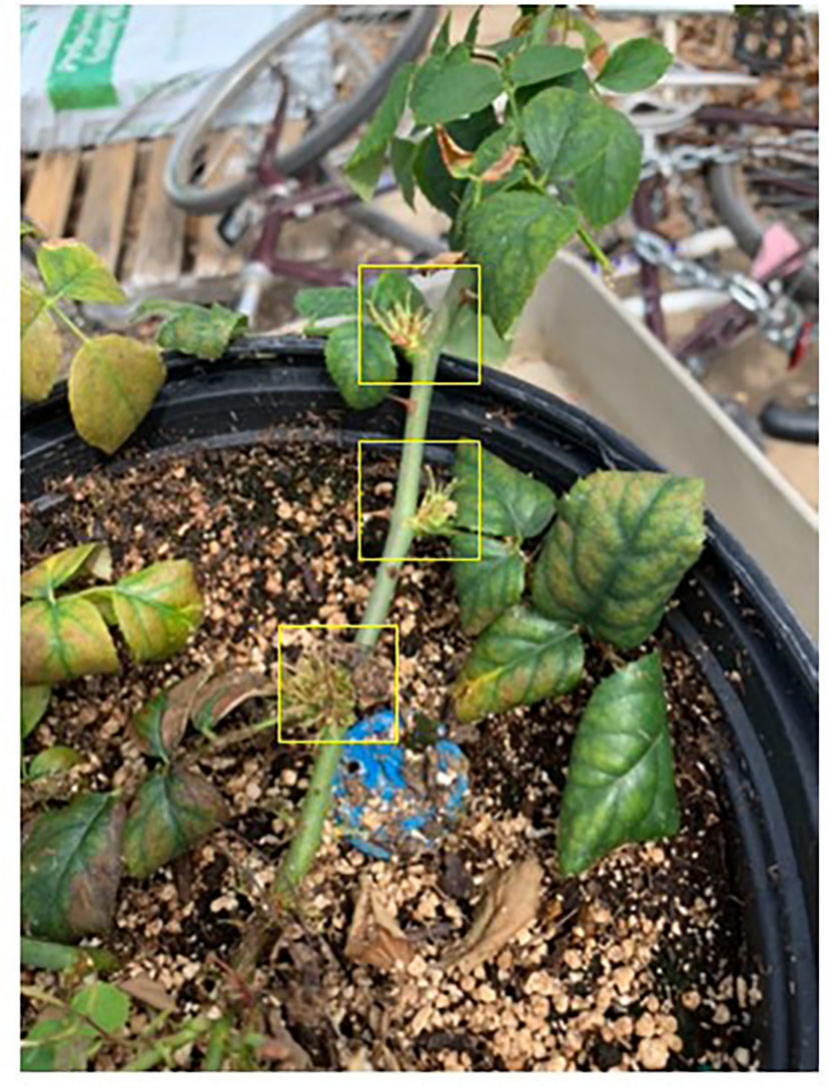
Herbicide induced symptoms on Roundup treated plants observed 21 days post treatment. This included proliferated shoots at nodes and chlorosis on new and old growth. Symptomatic damaged plant parts will not recover. Additionally, some necrosis was observed on new growth up to 30 days after treatment. Plants resumed normal appearing growth patterns about 30 days after the initial application (Shires, 2020).

**Figure 2:**
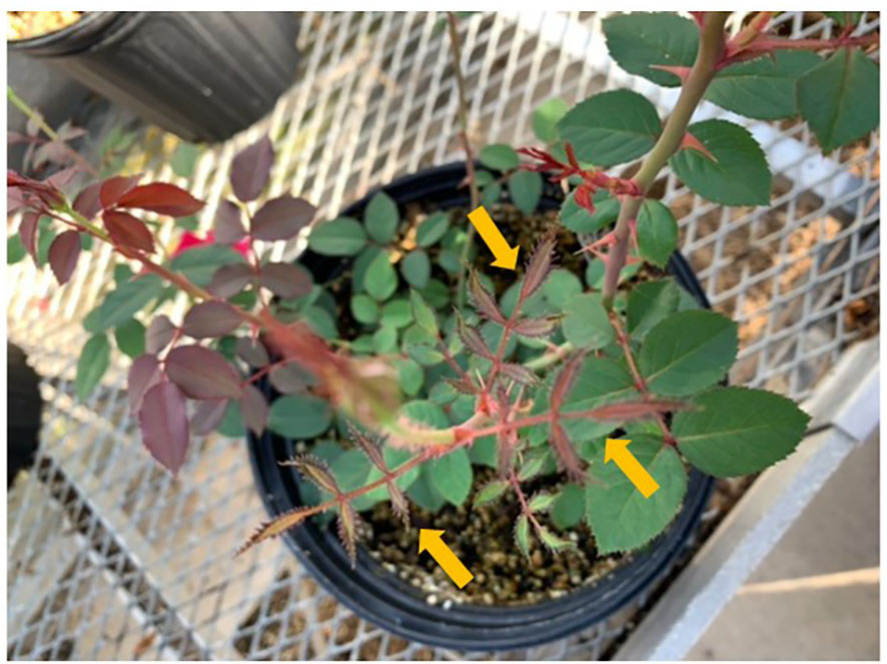
Typical symptoms caused by Weed-B-Gon include small, strapped leaves with abnormal leaf margins. Symptoms are visible, on the new growth, starting seven days post application. Plants appeared to resume normal new growth 30 days after treatment (Shires, 2020).

At day 7 post-treatment, we observed proliferation of small shoots on Glyphosate-treated plants (**Figure 1**) (Karlik and Flint). These plants began to exhibit leaf chlorosis at day 30 post-treatment. Roses exposed to WBG produced tiny leaves with abnormal margins at day 7 post-treatment (**Figure 2**). At day 30, WBG-exposed plants, had leaves of normal size, however, leaf margins remained abnormal. It should be noted that roses exposed to WBG did not change leaf color throughout the experiment.

### Herbicide treatment

**Figure 3:**
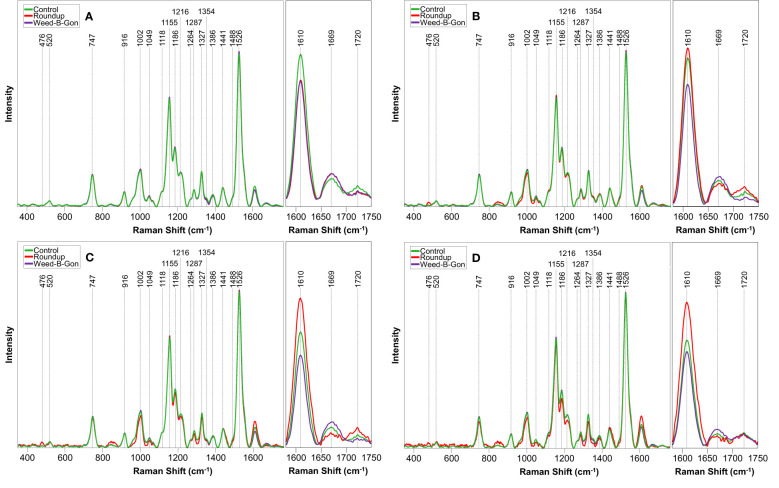
Raman spectra acquired from rose plants treated with RO water (control), Roundup, or Weed-B-Gon at **(A)** one day; **(B)** seven days; **(C)** 14 days; **(D)** 30 days after application. Inset: The spectral region 1580 cm^-1^ to 1750 cm^-1^ zoomed in for clarity. All spectra are normalized to the 1440 cm^-1^ peak.

At day 1 post-treatment, differences in the spectra of control and herbicide-treated plants were observed primarily in the 1610 to 1720 cm^-1^ region, **Figure 3** and **Table 1**. At this timepoint, the control spectra showed greater intensity in 1610 cm^-1^ and 1720 cm^-1^ peaks. The herbicide-treated plant spectra, conversely, had greater intensity than the control at 1669 cm^-1^ peak.

**Table 1:** Vibrational peak assignments for the Raman spectra of Rose leaves.

Peak (cm^-1^)	Vibrational mode	Assignment
476	Glycosidic ring	Carbohydrates (Kizil et al., 2002; Almeida et al., 2010)
520	ν(C-O-C) glycosidic	cellulose (Edwards et al., 1997)
740-747	γ(C–O-H) of COOH	pectin (Synytsya et al., 2003)
905-918	ν(C-O-C) in plane, symmetric	cellulose, lignin (Edwards et al., 1997)
1000	in-plane CH_3_ rocking of polyene	carotenoids (Schulz et al., 2005)
1048	ν(C-O)+ν(C-C)+δ(C-O-H)	cellulose, lignin (Edwards et al., 1997)
1118	Sym ν(C-O-C), C-O-H bending	cellulose (Edwards et al., 1997)
1157	C-C Stretching; v(C-O-C), v(C-C) in glycosidic linkages, asymmetric ring breathing	carotenoids (Schulz et al., 2005), carbohydrates (Wiercigroch et al., 2017)
1186	ν(C-O-H) Next to aromatic ring+σ(CH)	lignin (Mary et al., 2012; Agarwal, 2014)
1216	δ(C-C-H)	aliphatics (Yu et al., 2007), xylan (Agarwal, 2014)
1264	Guaiacyl ring breathing, C-O stretching (aromatic)	lignin (Cao et al., 2006)
1287	δ(C-C-H)	aliphatics (Yu et al., 2007)
1327	δCH_2_ Bending	aliphatics, cellulose, lignin (Edwards et al., 1997)
1354	δ(CH_2_)+δ(CH_3_)	aliphatics (Yu et al., 2007)
1386	δCH_2_ Bending	aliphatics (Yu et al., 2007)
1441	δ(CH_2_)+δ(CH_3_)	aliphatics (Yu et al., 2007)
1488	δ(CH_2_)+δ(CH_3_)	aliphatics (Yu et al., 2007)
1526	-C=C- (in plane)	carotenoids (Adar, 2017; Devitt et al., 2018)
1610	ν(C-C) Aromatic ring+σ(CH)	lignin (Agarwal, 2006; Kang et al., 2016)
1669	C=O Stretching, amide I	proteins (β-sheet) (Devitt et al., 2018)
1720	C=O Stretching	Esters, aldehydes, carboxylic acids and ketones (Colthup et al., 1990)

At day 7 post-treatment, the spectra of plants treated with each herbicide began to diverge. In the spectra of Roundup-treated plants, numerous changes throughout the spectrum were observed. Specifically, a new peak appeared at 476 cm^-1^. Additionally, small variations in intensity relative to the control spectrum were observed from 747 to 1526 cm^-1^ region. In the 1610 to 1720 cm^-1^ region, the spectra collected from Roundup-treated plants showed greater intensity than the spectra collected from both the control and WBG plants, whereas the spectra collected from WBG-treated plants exhibited the greatest intensity at 1669 cm^-1^. These same patterns were observed at day 14 post-treatment.

At day 30 post-treatment, while the general intensities throughout the spectra showed little relative changes, more alterations were observed in 1610 to 1720 cm^-1^ region. Specifically, all three treatments, Roundup, WBG and control, showed the same intensity of 1720 cm^-1^ peak. Additionally, the intensities of Roundup, WBG and control spectra changed relative to each other at the 1610 cm^-1^ peak. While Roundup-treated spectra continued to show the highest intensity at this band, the WBG and control spectra showed more similar intensities. Finally, the average quality of the Roundup spectra deteriorated to the point where the 476 cm^-1^ peak, previously observed at day 7 and day 14 post-treatment, was no longer distinguishable from the noise.

We then sought to determine whether Raman spectra acquired from control and herbicide-treated plants could be distinguished using multivariate methods. First, for the control and Roundup spectra, we built one PLS-DA model for each timepoint to distinguish these two categories from each other, **Table 2**. We found that these models enabled accurate identification of both WBG and Roundup-stresses (Chicco, 2017). We also found that PLS-DA enabled 92% accurate identification of control plants. These results demonstrated the RS could be used for the rapid, non-invasive detection of Roundup-associated changes to rose plants.

**Table 2:** Summary of model validation for the control vs. Roundup differentiation.

Days post-treatment	Validation Sample Size		True positive rate of control prediction	True positive rate of Roundup prediction
	Control	Roundup		
**Day 1**	35	38	85%	92%
**Day 14**	53	20	100%	100%
**Day 14**	24	53	70%	100%
**Day 30**	44	40	100%	97%

Next, we repeated this procedure to differentiate control and WBG spectra. It was found that developed PLS-DA models did not perform as well as the models discussed above. We obtained on average76% accuracy in differentiation between control and WBG spectra, **Table 3**. Furthermore, day 14 model performed very poorly, showing on average 59% accurate classification. However, models at day 7 and day 30 post-treatment performed significantly better showing 82% and 86% accuracy, respectively. These findings showed that RS could be used for the detection of WBG-associated stress in roses in a time-dependent manner.

**Table 3:** Summary of model validation for the control vs. WBG differentiation.

Days post-treatment	Validation Sample Size		True positive rate of control prediction	True positive rate of WBG prediction
	Control	WBG		
**Day 1**	46	30	84%	70%
**Day 14**	40	33	72%	93%
**Day 14**	48	26	45%	73%
**Day 30**	38	38	84%	89%

One may question the need for Raman diagnostics when it is not necessary to identify the specific herbicide that caused the damage. To answer this question, we combined spectra collected form WBG and Roundup treated plants at different stages of the plant vegetation and question the accuracy with which such spectra can be differentiated from the spectra collected from control plants at the same vegetation state, **Table 4**. We found that overall, such model demonstrates less accurate prediction on the healthy status of plants (control) compared to the herbicide damage. For instance, average accuracy of control plants ranges ~78%, whereas the accuracy of herbicide stress identification is within 85%, on average. We infer that the combined model (**Table 4**) provides lower accuracies due to different mechanisms of action of WBG and Roundup on plants. Although reported by this model accuracies (78% for control and 85% for the herbicide stress) are likely to be satisfactory for farmers, our data show that if higher accuracy is expected, herbicide-specific models should be used.

**Table 4:** Summary of model validation for control vs herbicides models.

Days post-treatment	Validation Sample Size		True positive rate of control prediction	True positive rate of herbicide prediction
	Control	Herbicides		
**Day 1**	109	49	73%	71%
**Day 14**	113	43	78%	86%
**Day 14**	75	86	76%	86%
**Day 30**	58	103	82%	89%

## Discussion

In plants, glyphosate concentrates in the meristems where it disrupts the shikimic pathway the prohibition of EPSP synthase (Sherwani et al., 2015). This decelerates plant growth and lowers protein expression. Roundup application also facilitates plant infection by soil-borne pathogens.

We found that spectra acquired from glyphosate-treated plants exhibited an increase in the intensity of ~1610 cm^-1^ peak at day 7, 14 and 30 post-treatment. We previously demonstrated that an increase in intensity of this peak was associated with plant infection by bacterial pathogens (Farber et al., 2019a; Sanchez et al., 2019b). Upon such infection, plants enhance production of *p*-coumaric acid that inhibits bacterial growth (Dou et al., 2021). This molecule is also used for lignin biosynthesis. Thus, producing p-coumaric acid species enhance lignification to limit bacterial propagation thought the plant. The observed in our current study increase in the intensity of ~1610 cm^-1^ peak suggest that glyphosate facilitated plant infection by pathogens present in soli (Hammerschmidt, 2018). This resulted in an increase in the synthesis of *p*-coumaric acid or similar aromatic compounds. Further experiments including mass spectrometry would be required to determine the exact biological origin of observed spectroscopic changes.

We also found that after day 1 post-treatment, the intensity of the 1669 cm^-1^ peak in the spectra acquired form glyphosate-treated plants was lower compared to the intensity of this peak in the spectra acquired from control or WBG-treated plants. This peak originates from amide I, a vibration of the backbone of proteins (Kurouski et al., 2015). A decrease in intensity of the 1669 cm^-1^ peak suggests that the total concentration of proteins is reduced in glyphosate-treated roses. These results are in a good agreement with the discussed above glyphosate-induced suppression of protein expression in plants.

Next, we found an increase in the intensity of 1720 cm^-1^ peak in the spectra collected from glyphosate-treated plants compared to the spectra acquired from control plants. This peak can be assigned to compounds with carboxyl groups, such as salicylic acid (Farber et al., 2019b; Farber et al., 2020b). This important signaling molecule is a product of the shikimate pathway (Gao et al., 2015). Thus, out results point on the accumulation of salicylic acid in plant leaves.

Unlike glyphosate, 2,4-D causes uncontrolled cellular division in plants that are exposed to this herbicide (Teale et al., 2006). This uncontrolled division is caused by cell wall plasticity, biosynthesis of proteins, and production of ethylene. MCPP is similar to 2,4-D, however, it targets auxin pathways causing elongated stems (Kelley and Riechers, 2007; Mithila et al., 2011).

We found that in the spectra collected WBG-treated plants, the intensity of the 1610 cm^-1^ peak was consistently lower compared to the intensity of this peak in the spectra acquired from control plants. These findings suggested that WBG lowered the concentration of *p*-coumaric acid or similar aromatic compounds in roses. Since *p*-coumaric acid is used in lignin biosynthesis (Amarowicz and Pegg, 2019), one can expect that uncontrolled cellular division in plants should drastically lower the concentration of such molecular analytes in plant tissues.

We also observed an increase in the intensity of 1669 cm^-1^ peak in the spectra acquired from WBG-treated plants compared to the intensity of this peak in the spectra acquired from control plants. As previously described, one effect for auxinic herbicides is dysregulation of auxin-regulated genes from said regulation, leading to an increase of gene products. Examples of these auxin-regulated genes include *Aux*/*IAA* family genes, GH3 proteins, and small auxin up RNAs (SAURs), which themselves are thought to code other short-lived small proteins (Kelley and Riechers, 2007). Increased intensity at this peak in the WBG spectra suggests that plants express more proteins, potentially due to WBG-associated gene dysregulation.

Finally, we found that at day 7, 14 and 30 post-treatments, the intensity of the 1720 cm^-1^ peak was weaker in WBG spectra compared to control spectra. These findings suggest that carboxyl-containing compounds could be metabolized by plants

Application of this Raman spectroscopy-based sensing approach could be limited due to the high capital cost of the spectrometers ($30,000 to $70,000). However, operational costs are very low. Therefore, if most cases, such testing can be implemented as the service provided to a farmer.

Finally, it is important to determine variability of the observed vibrational peaks in the spectra collected from different cultivars of roses. Such variabilities originate from differences in biochemical profiles of cultivars. Consequently, if biochemical changes among cultivars are greater that the magnitude of changes in plant biochemistry induced by herbicides, individual spectroscopic libraries will be required for each cultivar. At the same time, if the magnitude of changes in plant biochemistry induced by herbicides is greater than differences in biochemical profiles of different cultivars, the discussed above results can be used for all rose cultivars. Additional experiments are needed to disentangle these two possibilities. This work is currently in progress in our laboratory.

## Conclusions

Our results show that RS can be used to detect plant exposure to herbicides with high accuracy. We also found that RS could be used to differentiate between WBG and Roundup. We infer that this sensitivity arises from drastically different mechanisms of action of these two herbicides. These findings demonstrate that RS can be a powerful tool for detecting herbicide misuse on ornamental plant.

## Data availability statement

The raw data supporting the conclusions of this article will be made available by the authors, without undue reservation.

## Author contributions

CF, MS, and JU: investigation, data curation, and methodology. KO and DK: methodology, funding acquisition, and supervision. All authors contributed to the article and approved the submitted version.

## References

[B1] Adar F. (2017). Carotenoids - their resonance raman spectra and how they can be helpful in characterizing a number of biological systems. Spectroscopy 32, 12–20.

[B2] Agarwal U. P. (2006). Raman imaging to investigate ultrastructure and composition of plant cell walls: distribution of lignin and cellulose in black spruce wood (Picea mariana). Planta 224, 1141–1153. doi: 10.1007/s00425-006-0295-z 16761135

[B3] Agarwal U. P. (2014). 1064 nm FT-raman spectroscopy for investigations of plant cell walls and other biomass materials. Front. Plant Sci. 5, 1–12. doi: 10.3389/fpls.2014.00490 PMC4171993 25295049

[B4] Almeida M. R. Alves R. S. Nascimbem L. B. Stephani R. Poppi R. J. De Oliveira L. F. (2010). Determination of amylose content in starch using raman spectroscopy and multivariate calibration analysis. Anal. Bioanal. Chem. 397, 2693–2701. doi: 10.1007/s00216-010-3566-2 20213166

[B5] Amarowicz R. Pegg R. B. (2019). “Chapter one - natural antioxidants of plant origin,” in Advances in food and nutrition research. Eds. Ferreira I. C. F. R. Barros L. (New York, USA: Academic Press), 1–81. 10.1016/bs.afnr.2019.02.011 31445594

[B6] Benbrook C. M. (2016). Trends in glyphosate herbicide use in the united states and globally. Environ. Sci. Europe 28, 3–3. doi: 10.1186/s12302-016-0070-0 PMC5044953 27752438

[B7] Cao Y. Shen D. Lu Y. Huang J. (2006). A raman-scattering study on the net orientation of biomacromolecules in the outer epidermal walls of mature wheat stems (Triticum aestivum). Ann. Bot. 97, 1091–1094. doi: 10.1093/aob/mcl059 16533832 PMC2803402

[B8] Chicco D. (2017). Ten quick tips for machine learning in computational biology. BioData Min. 10, 35. doi: 10.1186/s13040-017-0155-3 29234465 PMC5721660

[B9] Colthup N. B. Daly L. H. Wiberley S. E. (1990). Introduction to infrared and raman spectroscopy (New York, USA: Academic Press).

[B10] Devitt G. Howard K. Mudher A. Mahajan S. (2018). Raman spectroscopy: an emerging tool in neurodegenerative disease research and diagnosis. ACS Chem. Neurosci. 9, 404–420. doi: 10.1021/acschemneuro.7b00413 29308873

[B11] Dou T. Sanchez L. Irigoyen S. Goff N. Niraula P. Mandadi K.. (2021). Biochemical origin of raman-based diagnostics of huanglongbing in grapefruit trees. Front. Plant Sci. 12, 680991. doi: 10.3389/fpls.2021.680991 34489991 PMC8417418

[B12] Edwards H. G. Farwell D. W. Webster D. (1997). FT raman microscopy of untreated natural plant fibres. Spectrochim. Acta A 53, 2383–2392. doi: 10.1016/S1386-1425(97)00178-9 9477578

[B13] Egging V. Nguyen J. Kurouski D. (2018). Detection and identification of fungal infections in intact wheat and sorghum hrain using a hand-held raman spectrometer. Anal. Chem. 90, 8616–8621. doi: 10.1021/acs.analchem.8b01863 29898358

[B14] Farber C. Bryan R. Paetzold L. Rush C. Kurouski D. (2020a). Non-invasive characterization of single-, double- and triple-viral diseases of wheat with a hand-held raman spectrometer. Front. Plant Sci. 11. doi: 10.3389/fpls.2020.01300 PMC7495046 33013951

[B15] Farber C. Kurouski D. (2018). Detection and identification of plant pathogens on maize kernels with a hand-held raman spectrometer. Anal. Chem. 90, 3009–3012. doi: 10.1021/acs.analchem.8b00222 29461798

[B16] Farber C. Sanchez L. Kurouski D. (2020b). Confirmatory non-invasive and non-destructive identification of poison ivy using a hand-held raman spectrometer. RCS Adv. 10, 21530–21534. doi: 10.1039/D0RA03697H PMC9054379 35518747

[B17] Farber C. Sanchez L. Pant S. Scheuring D. C. Vales M. I. Mandadi K.. (2021). Potential of spatially offset raman spectroscopy for detection of zebra chip and potato virus y diseases of potatoes (Solanum tuberosum). ACS Agric. Sci. Technol. 1, 211–221. doi: 10.1021/acsagscitech.1c00024

[B18] Farber C. Sanchez L. Rizevsky S. Ermolenkov A. Mccutchen B. Cason J.. (2020c). Raman spectroscopy enables non-invasive identification of peanut genotypes and value-added traits. Sci. Rep. 10, 7730. doi: 10.1038/s41598-020-64730-w 32382086 PMC7206150

[B19] Farber C. Shires M. Ong K. Byrne D. Kurouski D. (2019a). Raman spectroscopy as an early detection tool for rose rosette infection. Planta 250, 1247–1254. doi: 10.1007/s00425-019-03216-0 31222494

[B20] Farber C. Wang R. Chemelewski R. Mullet J. Kurouski D. (2019b). Nanoscale structural organization of plant epicuticular wax probed by atomic force microscope infrared spectroscopy. Anal. Chem. 91, 2472–2479. doi: 10.1021/acs.analchem.8b05294 30624904

[B21] Gao Q.-M. Zhu S. Kachroo P. Kachroo A. (2015). Signal regulators of systemic acquired resistance. Front. Plant Sci. 6. doi: 10.3389/fpls.2015.00228 PMC4394658 25918514

[B22] Gleason C. Foley R. C. Singh K. B. (2011). Mutant analysis in arabidopsis provides insight into the molecular mode of action of the auxinic herbicide dicamba. PloS One 6, e17245. doi: 10.1371/journal.pone.0017245 21408147 PMC3050828

[B23] Gupta S. Huang C. H. Singh G. P. Park B. S. Chua N.-H. Ram R. J. (2020). Portable raman leaf-clip sensor for rapid detection of plant stress. Sci. Rep. 10, 20206. doi: 10.1038/s41598-020-76485-5 33214575 PMC7677326

[B24] Hammerschmidt R. (2018). How glyphosate affects plant disease development: it is more than enhanced susceptibility. Pest Manage. Sci. 74, 1054–1063. doi: 10.1002/ps.4521 28067016

[B25] Henry W. B. Shaw D. R. Reddy K. R. Bruce L. M. Tamhankar H. D. (2004). Remote sensing to detect herbicide drift on crops. Weed Technol. 18, 358–368. doi: 10.1614/WT-03-098

[B26] Henry W. B. Shaw D. R. Reddy K. R. Bruce L. M. Tamhankar H. D. (2017). Remote sensing to detect herbicide drift on crops. Weed Technol. 18, 358–368. doi: 10.1614/WT-03-098

[B27] Kang L. Wang K. Li X. Zou B. (2016). High pressure structural investigation of benzoic acid: raman spectroscopy and x-ray diffraction. J. Phys. Chem. C. 120, 14758–14766. doi: 10.1021/acs.jpcc.6b05001

[B28] Karlik J. F. Flint M. L. Diseases and abiotic disorders of outdoor roses (American Phytopathological Society). Available at: https://www.apsnet.org/edcenter/apsnetfeatures/Pages/Roses.aspx (Accessed January 21 2021).

[B29] Kelley K. B. Riechers D. E. (2007). Recent developments in auxin biology and new opportunities for auxinic herbicide research. Pesticide Biochem. Physiol. 89, 1–11. doi: 10.1016/j.pestbp.2007.04.002

[B30] Kennard R. W. Stone L. A. (1969). Computer aided design of experiments. Technometrics 11, 137–148. doi: 10.1080/00401706.1969.10490666

[B31] Kizil R. Irudayaraj J. Seetharaman K. (2002). Characterization of irradiated starches by using FT-raman and FTIR spectroscopy. J. Agric. Food Chem. 50, 3912–3918. doi: 10.1021/jf011652p 12083858

[B32] Kurouski D. Van Duyne R. P. Lednev I. K. (2015). Exploring the structure and formation mechanism of amyloid fibrils by raman spectroscopy: a review. Analyst 140, 4967–4980. doi: 10.1039/C5AN00342C 26042229

[B33] Lu B. Dao P. D. Liu J. He Y. Shang J. (2020). Recent advances of hyperspectral imaging technology and applications in agriculture. Remote Sens. 12, 2659. doi: 10.3390/rs12162659

[B34] Mandrile L. Rotunno S. Miozzi L. Vaira A. M. Giovannozzi A. M. Rossi A. M.. (2019). Nondestructive raman spectroscopy as a tool for early detection and discrimination of the infection of tomato plants by two economically important viruses. Anal. Chem. 91, 9025–9031. doi: 10.1021/acs.analchem.9b01323 31265250

[B35] Mary Y. S. Panicker C. Y. Varghese H. T. (2012). Vibrational spectroscopic investigations of 4-nitropyrocatechol. Orient. J. Chem. 28, 937–941. doi: 10.13005/ojc/280239

[B36] Mehdizadeh M. Mushtaq W. Siddiqui S. A. Ayadi S. Kaur P. Yeboah S.. (2021). Herbicide residues in agroecosystems: fate, detection, and effect on non-target plants. Rev. Agricult. Sci. 9, 157–167. doi: 10.7831/ras.9.0_157

[B37] Mithila J. Hall J. C. William G. J. Kevin B. K. Dean E. R. (2011). Evolution of resistance to auxinic herbicides: historical perspectives, mechanisms of resistance, and implications for broadleaf weed management in agronomic crops. Weed Sci. 59, 445–457. doi: 10.1614/WS-D-11-00062.1

[B38] Payne W. Z. Kurouski D. (2021). Raman-based diagnostics of biotic and abiotic stresses in plants. a review. Front. Plant Sci. 11, 616672. doi: 10.3389/fpls.2020.616672 33552109 PMC7854695

[B39] Romero-Puertas M. C. Mccarthy I. Gómez M. Sandalio L. M. Corpas F. J. Del Río L. A.. (2004). Reactive oxygen species-mediated enzymatic systems involved in the oxidative action of 2,4-dichlorophenoxyacetic acid*. Plant Cell Environ. 27, 1135–1148. doi: 10.1111/j.1365-3040.2004.01219.x

[B40] Sanchez L. Ermolenkov A. Biswas S. Septiningshih E. M. Kurouski D. (2020). Raman spectroscopy enables non-invasive and confirmatory diagnostics of salinity stresses, nitrogen, phosphorus, and potassium deficiencies in rice. Front. Plant Sci. 11, 573321. doi: 10.3389/fpls.2020.573321 33193509 PMC7642205

[B41] Sanchez L. Farber C. Lei J. Zhu-Salzman K. Kurouski D. (2019a). Noninvasive and nondestructive detection of cowpea bruchid within cowpea seeds with a hand-held raman spectrometer. Anal. Chem. 91, 1733–1737. doi: 10.1021/acs.analchem.8b05555 30620572

[B42] Sanchez L. Pant S. Xing Z. Mandadi K. Kurouski D. (2019b). Rapid and noninvasive diagnostics of huanglongbing and nutrient deficits on citrus trees with a handheld raman spectrometer. Anal. Bioanal. Chem. 411, 3125–3133. doi: 10.1007/s00216-019-01776-4 30989272

[B43] Schulz H. Baranska M. Baranski R. (2005). Potential of NIR-FT-Raman spectroscopy in natural carotenoid analysis. Biopolymers 77, 212–221. doi: 10.1002/bip.20215 15674976

[B44] Sherwani S. I. Arif I. A. Khan H. A. (2015). “Modes of action of different classes of herbicides, herbicides, physiology of action, and safety,” in Herbicides: physiology of action and safety. Eds. Price A. Kelton J. Sarunaite L. (London, UK: IntechOpen Limited).

[B45] Shires M. K. (2020). Study of resistance to rose rosette disease utilizing field research, molecular methods, and transmission methods.

[B46] Synytsya A. Čopíková J. Matějka P. Machovič V. (2003). Fourier Transform raman and infrared spectroscopy of pectins. Carbohydr. Polym. 54, 97–106. doi: 10.1016/S0144-8617(03)00158-9

[B47] Tataridas A. Jabran K. Kanatas P. Oliveira R. S. Freitas H. Travlos I. (2022). Early detection, herbicide resistance screening, and integrated management of invasive plant species: a review. Pest Manag Sci. 78, 3957–3972. doi: 10.1002/ps.6963 35510308

[B48] Teale W. D. Paponov I. A. Palme K. (2006). Auxin in action: signalling, transport and the control of plant growth and development. Nat. Rev. Mol. Cell Biol. 7, 847–859. doi: 10.1038/nrm2020 16990790

[B49] Wiercigroch E. Szafraniec E. Czamara K. Pacia M. Z. Majzner K. Kochan K.. (2017). Raman and infrared spectroscopy of carbohydrates: a review. Spectrochim. Acta A 185, 317–335. doi: 10.1016/j.saa.2017.05.045 28599236

[B50] Yan Y. Ren J. Tschannerl J. Zhao H. Harrison B. Jack F. (2021). Nondestructive phenolic compounds measurement and origin discrimination of peated barley malt using near-infrared hyperspectral imagery and machine learning. IEEE Trans. Instrum. Measur. 70, 5010715. doi: 10.1109/TIM.2021.3082274

[B51] Yeturu S. Vargas Jentzsch P. Ciobotă V. Guerrero R. Garrido P. Ramos L. A. (2016). Handheld raman spectroscopy for the early detection of plant diseases: abutilon mosaic virus infecting abutilon sp. Anal. Methods 8, 3450–3457. doi: 10.1039/C6AY00381H

[B52] Yu M. M. Schulze H. G. Jetter R. Blades M. W. Turner R. F. (2007). Raman microspectroscopic analysis of triterpenoids found in plant cuticles. Appl. Spectrosc. 61, 32–37. doi: 10.1366/000370207779701352 17311714

