# Supplementary material for: Detection and differentiation of herbicide stresses in roses by Raman spectroscopy
Source: Front Plant Sci. 2023 Jun 5;14:1121012. doi: 10.3389/fpls.2023.1121012 (PMC10277736; doi:10.3389/fpls.2023.1121012)
Supplement: Supplementary file 1 [file DataSheet_1.pdf]

Supplemental Material

Tables S1-S2. Total number of spectra and size of validation dataset for each model calibrated in this study. Numbers in parentheses indicate how many spectra were taken from the overall sample to form the validation dataset.

Table number, Model	Total (Validation)		
	**Control**	**Roundup**	**Weed-B-Gon**
**1, Day 1**	109 (35)	106 (38)	n/a
**1, Week 1**	105 (53)	112 (20)	n/a
**1, Week 2**	119 (24)	110 (53)	n/a
**1, Month**	118 (44)	114 (40)	n/a
**2, Day 1**	109 (46)	n/a	115 (30)
**2, Week 1**	105 (53)	n/a	112 (20)
**2, Week 2**	117 (48)	n/a	115 (26)
**2, Month**	118 (38)	n/a	106 (38)

Model	Class	Total (Validation)
	**Control**	**Herbicide**
**Day 1**	241 (109)	224 (49)

**Week 1**	238 (113)	223 (43)
**Week 2**	253 (75)	221 (86)
**Month 1**	253 (58)	223 (103)

Table S3. Prediction results of the differentiation of spectra acquired from control and Roundup-treated roses at one month after application, divided into calibration and validation sets using the Kennard-Stone method. CV - Cross-validation. V – validation. C – Control. R – Roundup. TPR - True Positive Rate (accuracy).

**Model**	Validation Sample Size (C,R)	**TPR Control**	**TPR Roundup**
**Month CV**	117,37	96%	94%
**Month V**	1,77	100%	100%

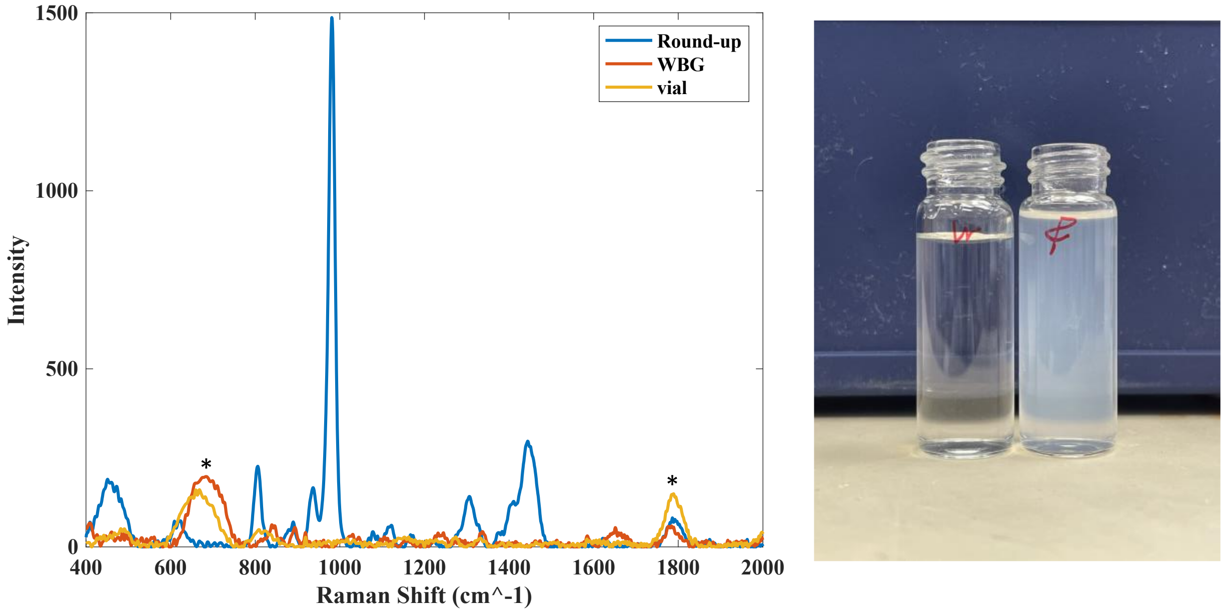

Figure 1. Reference Raman spectra of round-up and WBG solutions.
